# Supplementary material for: Stereotactic Surgery for Treating Intractable Tourette Syndrome: A Single-Center Pilot Study
Source: Brain Sci. 2022 Jun 28;12(7):838. doi: 10.3390/brainsci12070838 (PMC9313141; doi:10.3390/brainsci12070838)
Supplement: Supplementary file 1 [file brainsci-12-00838-s001.zip › brainsci-1738139-supplementary.pdf]

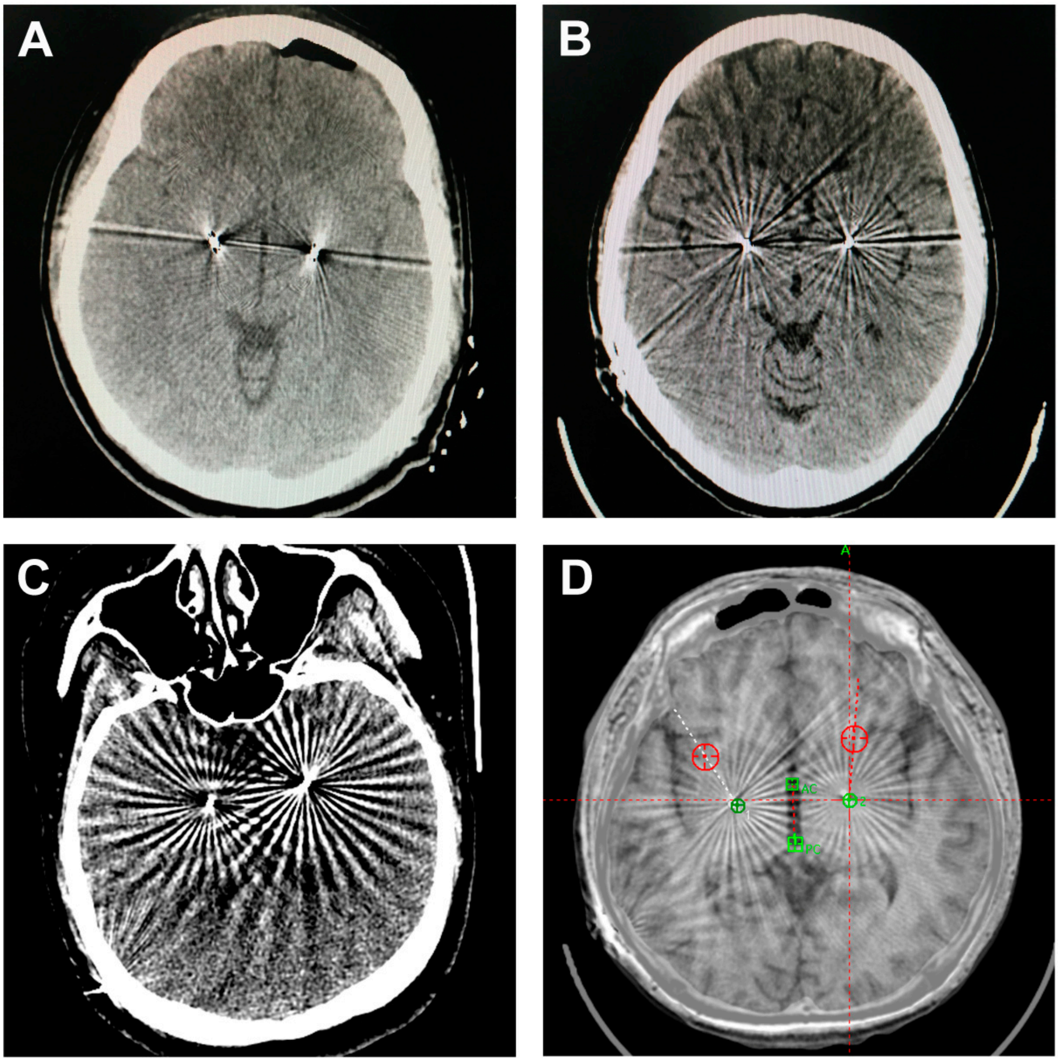

Figure S1. Location of the leads on postoperative imaging data. A-C, Case 9-11, computed tomography (CT) scan of the brain after lead implantation, demonstrating the bilateral leads in the GPi; D, Case 12, fusion of the postoperative CT with preoperative magnetic resonance imaging (MRI), demonstrating the bilateral leads in the GPi.
